# Swimming performance of *Bradyrhizobium diazoefficiens* is an emergent property of its two flagellar systems

**DOI:** 10.1038/srep23841

**Published:** 2016-04-07

**Authors:** J. Ignacio Quelas, M. Julia Althabegoiti, Celia Jimenez-Sanchez, Augusto A. Melgarejo, Verónica I. Marconi, Elías J. Mongiardini, Sebastián A. Trejo, Florencia Mengucci, José-Julio Ortega-Calvo, Aníbal R. Lodeiro

**Affiliations:** 1 Instituto de Biotecnologia y Biologia Molecular (IBBM) Facultad de Ciencias Exactas, UNLP-CONICET Argentina; 2 Instituto de Recursos Naturales y Agrobiología de Sevilla (IRNAS)-CSIC Spain; 3 Departamento de Ciencias Básicas, Facultad de Ingeniería, UNLP Argentina; 4 Facultad de Matemáticas, Astronomía y Física (FAMAF)-UNC e IFEG-CONICET Argentina; 5 Servei de Proteòmica i Biologia Estructural, Universitat Autònoma de Barcelona, Bellaterra Spain

## Abstract

Many bacterial species use flagella for self-propulsion in aqueous media. In the soil, which is a complex and structured environment, water is found in microscopic channels where viscosity and water potential depend on the composition of the soil solution and the degree of soil water saturation. Therefore, the motility of soil bacteria might have special requirements. An important soil bacterial genus is *Bradyrhizobium*, with species that possess one flagellar system and others with two different flagellar systems. Among the latter is *B. diazoefficiens*, which may express its subpolar and lateral flagella simultaneously in liquid medium, although its swimming behaviour was not described yet. These two flagellar systems were observed here as functionally integrated in a swimming performance that emerged as an epistatic interaction between those appendages. In addition, each flagellum seemed engaged in a particular task that might be required for swimming oriented toward chemoattractants near the soil inner surfaces at viscosities that may occur after the loss of soil gravitational water. Because the possession of two flagellar systems is not general in *Bradyrhizobium* or in related genera that coexist in the same environment, there may be an adaptive tradeoff between energetic costs and ecological benefits among these different species.

The earliest microbiologists were astonished when they observed bacterial motility under their rudimentary microscopes, as this trait undoubtedly indicated to them that such tiny entities are living beings^1^. Since then, at least seven different types of bacterial motility have been described^2^, among which swimming and swarming are propelled by flagella. The paradigm of bacterial swimming in liquid medium is the run-tumble (RT) model that is described in *Escherichia coli* and *Salmonella*^3^. In these bacteria, the peritrichous flagella switch rotation from counter clockwise (CCW) to clockwise (CW) direction. When flagella co-ordinately rotate CCW, the filaments form a bundle, and the cell advances in a run until it is interrupted at the switching of flagellar rotation to the CW sense, which disrupts the bundle and produces a tumble. In the presence of attracting/repellent chemicals such as nutrients, toxins or environmental signals, bacterial swimming is guided by the chemotaxis system, which couples the detection of concentration gradients to the frequency of changes in flagellar rotational sense or speed allowing the bacteria to perceive and respond to such gradients in real time^3^,^4^,^5^. Hence, bacterial swimming is a characteristic biased random walk with a short memory of the previous direction of motion.

Recent investigations have described alternatives to the RT model^6^. Some soil bacteria, such as *Ensifer meliloti*, *Rhizobium lupini*, and *R. leguminosarum*, possess peritrichous flagella that rotate in the CW direction only. In these bacteria, runs are produced when flagella rotate co-ordinately in a bundle, while a tumble occurs when individual flagella stop or slow down, causing bundles to fall apart^7^,^8^,^9^. *Caulobacter crescentus* possesses a monotrichous polar or subpolar flagellum, and its switch from the CW to CCW rotational sense cause reversals of swimming with steep changes in direction caused by Brownian effects^10^. In addition to this run-reverse (RR) swimming, a three-step behaviour named run-reverse-flick (RRF) was recently described in aquatic bacteria^11^,^12^, in which the torsion of the flagellar-cell body longitudinal axis is produced upon reversal, leading to reorientation at an approximately right angle^12^.

*Bradyrhizobium diazoefficiens* USDA 110, a soil α-proteobacterium that fixes atmospheric N_2_ in symbiosis with soybean, possesses two different flagellar systems. These systems are described as subpolar flagella based on the *Salmonella* model^13^, but more careful studies with various α-, β-, and γ-proteobacteria indicated that the primary system is related to flagella of *C. crescentus*, *Rhodopseudomonas palustris* and *Rhodospirillum rubrum*, while the secondary system is related to flagella of *E. meliloti*, *Mesorhizobium loti* and *R. etli*^14^. Dual flagellar systems have been found in several species, being *Vibrio* spp. the most studied. However, these γ-proteobacteria are taxonomically distant to *B. diazoefficiens*, and their flagellar systems seem unrelated^14^. Previous reports in bacteria with dual flagellar systems indicate that in general, the primary system is specific for swimming in liquid medium and the other −lateral− for swarming on wet surfaces^15^,^16^. However, planktonic cells of *B. diazoefficiens* may produce both flagellar systems simultaneously in liquid medium^13^,^17^,^18^,^19^, with *Shewanella putrefaciens* being the only other known example with this capability^20^.

Studies of *B. diazoefficiens* mutants lacking either the primary or secondary flagellar filaments did not allow for the assignment of clearly distinct functions to each one in swimming, swarming or competition for soybean nodulation^18^,^19^. This situation is somewhat enigmatic, as flagella are complex structures that are encoded in a core set of 24 structural genes^21^, requiring another set of similar size for regulation and consuming much energy in rotation. Therefore, the simultaneous expression of two different flagellar systems in planktonic state seems justified if it provides some adaptive advantage. With the aim of clarifying the roles of the two flagellar systems of *B. diazoefficiens* here, we intended to assess their contributions to swimming performance in liquid and semisolid media, attachment to glass, and chemotaxis.

## Results

### Structure and taxonomic relationships of *B. diazoefficiens* flagellar systems

**Figure 1:**
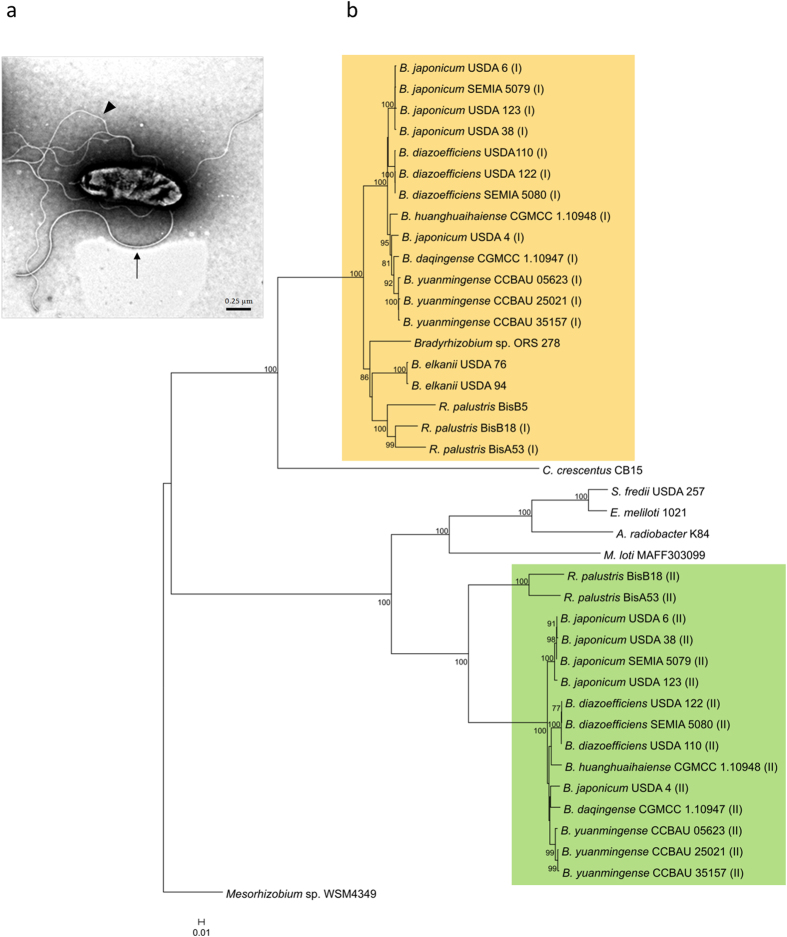
Flagellar systems of *B. diazoefficiens*. (**a**) Transmission electron micrograph of a planktonic cell that was grown in liquid AG medium, with its subpolar flagellum (*arrow*) and at least five lateral flagella, some of which are partially bundled (*arrowhead*). The contrast was enhanced to better appreciate flagellar filaments. Scale bar: 0.25 μm. (**b**) Neighbour-joining phylogeny based on concatenated sequences of FlhA, FlhB, FliF, FliG and FliP of main *Bradyrhizobium* species showing the primary (I) and secondary (II) flagellar systems. Bootstrap values higher than 70 are indicated at the nodes. Scale bar, 0.01 amino acid substitutions per site.

The cellular disposition of *B. diazoefficiens* flagellar systems is shown in Fig. 1a, where a planktonic cell from liquid culture is observed displaying its monotrichous constitutive subpolar flagellum with a short and thick filament and its inducible lateral flagella with filaments longer, thinner, wavy and sometimes associated in bundles. The subpolar flagellar filaments are composed of the 68-kDa flagellins FliC1, FliC2, FliC3 and FliC4 (bll5843-bll5846) and the lateral ones of the 34-kDa flagellins LafA1 (bll6866) and LafA2 (bll6865) (Supplementary Information Appendix).

The subpolar flagellar genes are dispersed in various gene clusters, and their taxonomic relationships are not congruent to those of lateral flagellar genes, which are grouped in a single cluster. These features suggest that the lateral system was acquired by horizontal gene transfer^14^. To test this hypothesis, we looked for flanking insertion sequences or significant differences in the GC-skew^22^ in or around the lateral flagellar gene cluster, but no such evidence could be found. To go further in this taxonomic characterization, we generated a cladogram with concatenated FlhA, FliG, FliP, FlhB and FliF^14^ using recently reported genomic sequences of the main soybean-nodulating *Bradyrhizobium* species, as well as from *R. palustris*, *C. crescentus*, *E. meliloti*, *Sinorhizobium fredii*, *Agrobacterium radiobacter*, *Mesorhizobium* sp. and *M. loti*. (Fig. 1b). We found that, as previously reported, primary and secondary flagellar systems were clearly separated. The subpolar flagella are similar to those of *C. crescentus*, while the lateral flagella are similar to those of *S. fredii*, *E. meliloti*, *A. radiobacter* and *M. loti*. A very close relationship was observed with *R. palustris*, in which strains BisB18 and BisA53 have both flagellar systems, while strain BisB5 has only the primary flagellar system. Regarding *Bradyrhizobium* flagella, the cladogram also grouped the flagella belonging to the different species of this genus, except those of *B. japonicum* USDA 4, whose primary and secondary systems both appeared intermingled among *B. huanghuaihaiense*, *B. daqingense* and *B. yuanmingense*. However, *B. japonicum* USDA 4 was already observed as unrelated to other *B. japonicum* strains^23^. In addition, the average nucleotide identity (ANI) values between genomes of USDA 4 and *B. japonicum* USDA 6^T^ was 90.43, and between USDA 4 and *B. diazoefficiens* USDA 110^T^ was 90.59, suggesting that USDA 4 should not be considered as any of these species.

Interestingly, the presence of two flagellar systems is not universal in the soybean-nodulating *Bradyrhizobium*: *B. elkanii* USDA 76 and USDA 94 do not possess the secondary system. However, these strains were more closely related to the three strains of *R. palustris*, two of which possess a secondary system. In addition, we could find neither *flhB* nor *fliF* in *B. liaoningense*, whereby this species could not be included in the cladogram. As in *B. diazoefficiens*, clear evidence of horizontal gene transfer of the secondary system was not observed in either *B. japonicum* or *R. palustris*.

### Swimming performance of *B. diazoefficiens* USDA 110 is complex and differs for each flagellar system

A total of 346 swimming cells of *B. diazoefficiens*, including wild-type and two derivative mutants, one devoid of lateral flagellar filaments (Δ*lafA*) and the other lacking the subpolar flagellar filament (Δ*fliC*), were tracked at 30 frames s^−1^ in videos of 5–6 s of duration. Because bacterial cells could be tracked only while remaining in the focal plane, the duration of individual trajectories varied within videos. The mean durations (±confidence intervals, α = 0.05) for each strain were 3.44 ± 0.17 s (*n* = 121) for the wild-type, 3.22 ± 0.20 s (*n* = 110) for Δ*lafA* and 4.26 ± 0.09 s (*n* = 115) for Δ*fliC*. Hence, the total time recorded was 6.93 min for the wild-type, 5.90 min for Δ*lafA* and 8.16 min for Δ*fliC*.

**Figure 2:**
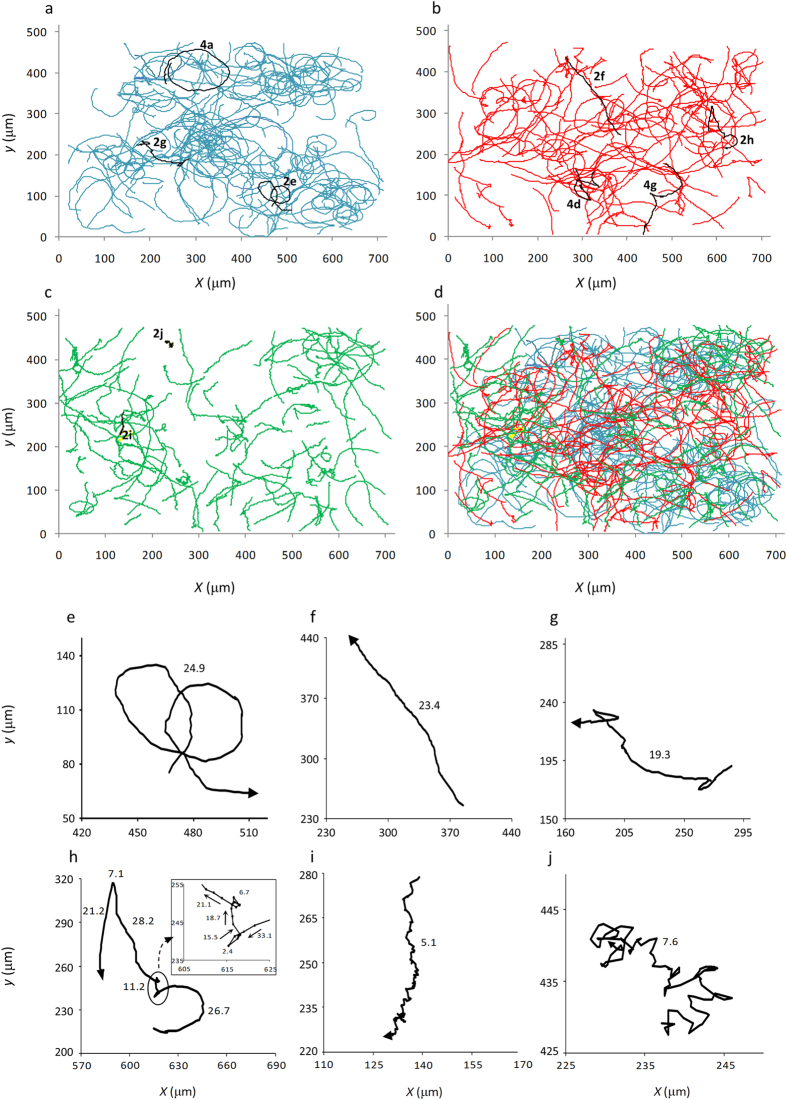
Overview of the trajectory shapes. All of the trajectories that were analyzed in this study are shown for wild-type ((**a**) *n* = 121, *blue*), Δ*lafA* ((**b**) *n* = 110, *red*) and Δ*fliC* ((**c**) *n* = 115, *green*). Highlighted in *black* are the trajectories that were selected in Figs 2e–j, 4a,d,g and numbered according to the figure number in which they appear. In (**d**), all of the trajectories that were obtained in 11 different observations from 7 independent cultures are shown together with the same colour codes as in panels (**a**–**c**). Examples of trajectory shapes are shown in (**e**–**j**): circular ((**e**) wild-type), linear ((**f**) Δ*lafA*), irregular with RR changes in direction ((**g**) wild-type), irregular with RR and RRF changes in direction ((**h**) Δ*lafA*), rough ((**i**) Δ*fliC*) and diffusive ((**j**) Δ*fliC*). In the *inset* of panel (**e**), an enlargement of the run-reverse-flick event is shown, with the position of the cell every 33 ms indicated by dots and the swimming direction indicated by arrows. Coordinate axis values in (**e**–**j**) correspond to those of (**a**–**d**). The numbers next to the trajectories indicate the mean speeds in micrometers per second (μm·s^−1^).

The trajectory shapes that were obtained were complex, all of which are shown together in Fig. 2a–d, where some biases among the different strains may be noticed. Some trajectories were smooth, including closed circles (Figs 2e and 4a), open curves, and linear paths with almost no curvature (Fig. 2f). Others were irregular, with changes in direction in the RT, RR and RRF modes (Fig. 2g,h), as well as tight RT in Δ*fliC* (Fig. 2i,j). In some cases, these features were combined in a single trajectory, as in the example of Fig. 2h. According to descriptions in other species^24^,^25^, this trajectory might have started with the bacterium swimming backward, reversing with a steep fall in swimming speed (from 33.1 to 2.4 μm·s^−1^), starting again in the forward direction, which provoked a “flick” at low speed (15.5–18.7 μm·s^−1^), and finally resuming swimming at normal speed with RR/RT changes of direction. The average run speeds (μm·s^−1^ ± confidence intervals, α = 0.05) in smooth trajectories were 28.8 ± 1.3 (*n* = 48) for the wild-type, 26.9 ± 2.0 (*n* = 50) for Δ*lafA*, and 17.2 ± 0.8 (*n* = 28) for Δ*fliC*.

**Figure 3:**
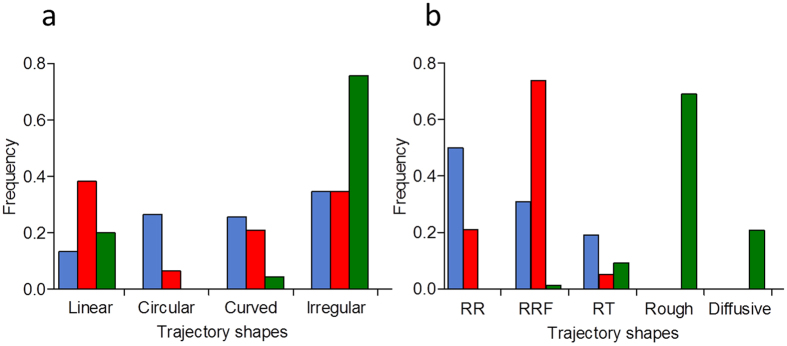
Frequencies of the different trajectory shapes in the wild-type (*blue*), Δ*lafA* (*red*) or Δ*fliC* (*green*). (**a**) Sorting in linear, circular, curved or irregular. (**b**) Sorting of irregular trajectories according to the predominant change in direction mode.

Despite complexity, the trajectory shapes seemed to be differently distributed among the wild-type and the mutants (Fig. 2a–d). We intended to sort the trajectories into linear, circular, curved and irregular (Fig. 3a) and observed that irregular trajectories were much more frequent in Δ*fliC*. In addition, the frequency histogram showed that the frequencies of curved and irregular trajectories were similar between the wild-type and Δ*lafA*, but there were clear biases toward circular trajectories in the wild-type and toward linear trajectories in Δ*lafA*. We sorted irregular trajectories according to their predominant changes in direction in RR, RRF and RT in the wild-type and Δ*lafA* and tight RT in Δ*fliC*, which we further divided into rough (Fig. 2i) and diffusive (Fig. 2j). Again, we found different biases predominating RR in the wild-type, RRF in Δ*lafA*, and rough and diffusive in Δ*fliC* (Fig. 3b). These biases agree with the type of flagella of each mutant, as RR and RRF changes in direction are described in species with single polar or subpolar flagella^10^,^11^,^12^, while RT changes in direction are more typical of bacteria with peritrichous flagella^3^,^4^,^7^,^8^,^9^. The fact that the wild-type has more RR/RRF than RT changes in direction might be explained by the low efficiency of lateral flagella for swimming in liquid medium. However, there were ambiguous cases, as wide open curves or circles punctuated by RR or RRF changes in direction (Supplementary Fig. S2), which introduced arbitrariness into the sorting of Fig. 3.

### Trajectory Index

**Figure 4:**
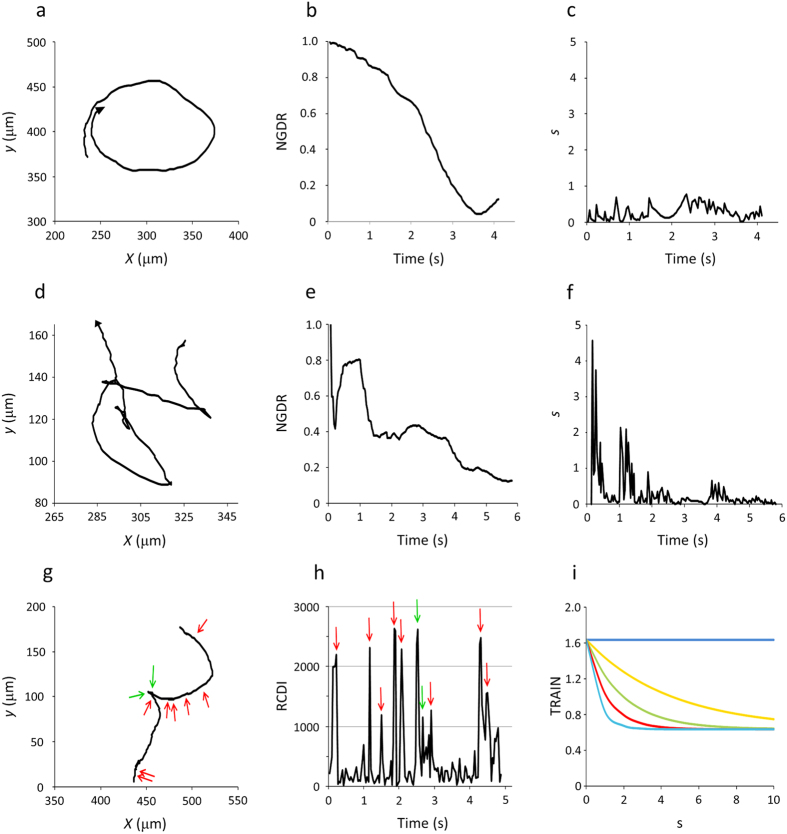
Illustration of different components of the trajectory index (*TRAIN*). In (**a**,**d**), two trajectories with low *NGDR* are compared. (**a**) Smooth circular trajectory (wild-type, *NGDR* = 0.12). (**d**) Irregular trajectory (Δ*lafA*, *NGDR* = 0.13). In each one, the rate of change of *NGDR* over time is different, being mild in the smooth trajectory (**b**) or steep in the irregular trajectory (**e**), giving rise to low (**c**) or high (**f**) absolute values of the maximal slope (*s*). (**g**) A trajectory of Δ*lafA* with an RRF showing the two changes in direction (*green arrows*) that were correctly predicted as *RCDI* > 1,000 (**h**), although there were 8 false positives in positions with *RCDI* > 1,000 but no apparent change in direction (*red arrows*). (**i**) Generic variation of *TRAIN*. In the graph, the values of <cos *α*> and *c* were fixed at 0.8 and 2, respectively, and *NGDR* was considered as 1.0 (*blue*), 0.8 (*orange*), 0.6 (*green*), 0.4 (*red*) or 0.2 (*light blue*). Hence, these curves depict the variation of *TRAIN* with *NGDR* and *s* when <cos *α*> and *c* are fixed, but because the curves were obtained from *y* = *a* + *b*^*x*^, they can also illustrate how *TRAIN* varies with <cos *α*> and *c* when *NGDR* and *s* are fixed. The coordinate values in (**a**,**d**,**g**) correspond to those of Fig. 2a–d.

To find an objective and quantitative classification of trajectories, we built a trajectory index (*TRAIN*) based on known quantitative descriptors of path shape. The curvature of smooth trajectories may be estimated by their radius of curvature or may be reflected in the net-to-gross displacement ratio (*NGDR*)^26^, which relates the net distance between two points in the *x*-*y* coordinate space to the gross distance that is actually travelled between these same points, according to the expression:


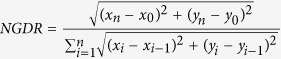


where *x* and *y* are the coordinates of the position, *i* represents the *i*-th position, *0* represents the initial position, and *n* is the number of successive positions that were considered. Therefore, persistently circular trajectories tend to present *NGDR* values between start and end points near 0, while linear trajectories have *NGDR* values near 1. With this criterion, in Fig. 3, we classified the smooth trajectories as circular when their *NGDR* were <0.25, linear when their *NGDR* were >0.75, and curved when their *NGDR* were in between those values. The *NGDR* has the advantage over the radius of curvature that it can be used with any trajectory shape, curved or not. In addition to circular trajectories, those with significant changes in direction (low persistence) may also give *NGDR* values near 0 between start and end points. The persistency of trajectories may be evaluated through the rate of change of *NGDR*, which is low in smooth trajectories. In contrast, in trajectories with low persistence, sudden changes in direction are reflected in high absolute values of the slope of *NGDR* against the time of successive positions (Fig. 4a–f). Hence, the maximum absolute value of the slope of *NGDR* (*s*):


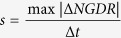


registered in a trajectory is a good indicator of its persistence.

All trajectories, either smooth or irregular, showed changes in direction frame by frame. Most of these changes in direction were due to translational and rotational diffusion, with average angle of 18 ± 3°, but some of them were due to active switching of bacterial motion and had higher angles. Therefore, we calculated the cosine of the angle (*α*_*i*_) between frames:


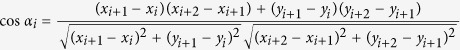


and obtained its average extended to all positions of a given trajectory (<cos *α*>). Next, we looked for significant changes in direction using the relative change in direction index (*RCDI*) that was provided by the software^26^. We observed that in general, *RCDI* values higher than 1,000 corresponded to *x-y* positions in which cos *α*_*i*_ was lower than <cos *α*> minus two standard deviations, suggesting that peaks of *RCDI* higher than 1,000 would identify significant changes in direction^27^. However, when we intended to confirm this criterion by the visual inspection of each trajectory, we found several false positives that we had to eliminate before estimating the actual number of significant changes in direction, *c* (Fig. 4g,h). Together, <cos *α*> and *c* seemed to be good indicators of the irregularity of trajectories.

With all these descriptors, we defined the trajectory index (*TRAIN*) as follows:


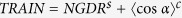


The first term, which may take values between 0 and 1 because 0≤ *NGDR* ≤1 and *s* ≥ 0, represents the circularity/linearity and persistence of the trajectory, while the second term, whose similarly is between 0 and 1 because 0 ≤ <cos *α*> ≤ 1 and *c* is an integer ≥ 0, represents the irregularity of the trajectory. In Fig. 4i, we show how *TRAIN* varies in response to *s* or *c* for different values of *NGDR* or <cos *α*>. The *NGDR*, *s*, <cos *α*>*, c* and *TRAIN* values for the examples chosen in Figs 2,4 are shown in the Supplementary Table S3.

Hence, 0 ≤ *TRAIN* ≤ 2 approaches 0 for highly irregular trajectories, 1 for circular trajectories or 2 for linear trajectories. Thus, average ± standard deviation (SD) *TRAIN* values distinguished irregular trajectories with an RRF mode of change in direction (0.53 ± 0.38; *n* = 40) from smooth circular (1.04 ± 0.11; *n* = 40) and linear (1.79 ± 0.23; *n* = 58), without differences among the wild-type and Δ*lafA*. The swimming of Δ*fliC* was different, with two main trajectory shapes: rough (Fig. 2i), with very short runs and high persistence (*TRAIN* = 0.43 ± 0.29; *n* = 60), and diffusive (Fig. 2j), with very low persistence (*TRAIN* = 0.05 ± 0.12; *n* = 18) −see also Figs 2d and 3. Although curved (non-circular) trajectories or irregular trajectories with few changes in direction had intermediate *TRAIN* values, we considered that the frequency distribution of *TRAIN* values is a more objective estimate of the trends of trajectory shapes in each strain than the classification that is made by eye, or assisted with *NGDR* (see also Supplementary Fig. S2).

### *TRAIN* frequency histograms characterized swimming behaviour of the wild-type and the mutants

**Figure 5:**
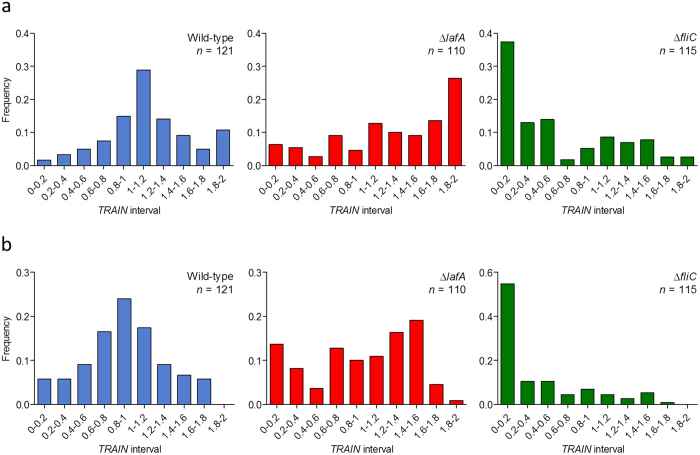
Frequencies of trajectory shapes in the three strains assessed by the trajectory index (*TRAIN*) values, which tend to be low in irregular trajectories, middle in circular trajectories, and high in linear trajectories. (**a**) Non-corrected *TRAIN* values. (**b**) *TRAIN* values corrected with the trend line equations (cf. Fig. 6) to carry all durations to 6 s (see text for details). Standard deviations: for wild-type, non-corrected: 0.42; corrected: 0.41. For Δ*lafA*, non-corrected: 0.59; corrected: 0.57. For Δ*fliC*, non-corrected: 0.57; corrected: 0.53.

The frequency histograms of *TRAIN* for the different strains are shown in Fig. 5a. In the wild-type, *TRAIN* frequency histogram had a bell-shaped distribution that was centred at intermediate values, in agreement with a high frequency of circular trajectories. In the mutants, *TRAIN* frequency histograms were biased to the limits: towards the upper (linear trajectories) in Δ*lafA* and towards the lower (highly irregular trajectories) in Δ*fliC*. Furthermore, wild-type trajectories were more like each other than the Δ*lafA* or Δ*fliC* mutants, as indicated by their coefficients of variation (37.2%, 46.0%, and 97.7%, respectively).

**Figure 6:**
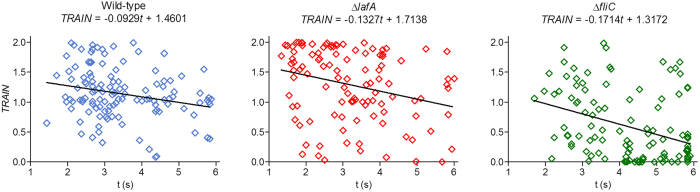
Correlations between trajectory duration and trajectory index (*TRAIN*). Shown are the individual trajectory values (*open diamonds*) and trend lines (*black*).

Because both the circularity and the probability of finding significant changes in direction depend in some way on the duration of the analyzed trajectory, we looked for a correlation between *TRAIN* values and trajectory durations (Fig. 6). We found negative correlations (*r* values −0.24, −0.28 and −0.36 for the wild-type, Δ*lafA* and Δ*fliC*, respectively, all them significant with α = 0.05), which lead us to question its influence on the distributions of *TRAIN* values in the frequency histograms. In particular, linear trajectories in Δ*lafA* seemed shorter than the others (see the accumulation of dots at the upper left corner in the Δ*lafA* panel of Fig. 6). Thus, the proportion of smooth linear trajectories might be overestimated, and the trends that are suggested by the frequency histograms (Fig. 5a) might be an artefact. To evaluate this possibility, we used the slopes of the linear tendencies to extrapolate all *TRAIN* values to 6.0 s, the duration of the longest trajectory. These corrected *TRAIN* values were in general lower than the original ones, but when we plotted their frequencies for each strain, we observed that the trends obtained above and the SD were not modified (Fig. 5b), indicating no artificial biases. These results agree with previous observations in *S. putrefaciens* CN-32, where lateral flagella stabilize swimming, and a mutant lacking the polar flagellum moves irregularly^20^.

The different *TRAIN* biases among the three strains and the lower coefficient of variation in the wild-type suggest an epistatic relationship between the *fliC1234* and *lafA12* gene groups. To determine their interaction, we formed 10 sets, each containing 20 randomly chosen trajectories of each strain, and performed a factorial analysis of variance. We obtained an average *F*_*1,57*_ value of 12.59 in a range of 8.05–26.67 for the *fliC*-*lafA* interaction, all them statistically significant with α = 0.01.

### Attachment to glass slides is interfered by lateral flagella

The observation that the wild-type swam mainly in circles, while in Δ*lafA*, the trajectories were predominantly linear, prompted us to examine whether these differences may be parallel to differences in attachment to the surface of the glass slides used for tracking. To this end, we incubated the bacterial cells on the glass slides for 1 h, washed the non-adherent cells, and made an estimation of the mean number of attached cells ( ± confidence intervals, α = 0.05) as described in the Methods section. These numbers were 47.4 ± 12.1 for the wild-type, 144.2 ± 52.9 for Δ*lafA*, 11.5 ± 5.6 for Δ*fliC*, and 12.6 ± 9.1 for Δ*fliC*/Δ*lafA*. Thus, lateral flagella were not required, but seemed to interfere with the subpolar flagellum for attachment to glass.

### Only the subpolar flagellum responded chemotactically to glutamate and succinate

Previously, the chemotaxis of the bradyrhizobial strains USDA 110, 10 K and 643 b was studied with a wide variety of attractant substances (chemoattractants), which presumably are nutrients and signals present in the soybean root exudates^28^,^29^. Both reports are coincident in that glutamate and succinate are the strongest chemoattractants. In addition, mannitol and arabinose were characterized as poor chemoattractants in USDA 110 28 but as strong chemoattractants in 10 K and 643 b^29^. Nevertheless, we were interested in these two sugars because we previously observed that they condition the expression of the lateral flagella^19^. Hence, we measured the chemotaxis towards the four compounds with the chemoattractant-in-capillary assay^27^.

**Figure 7:**
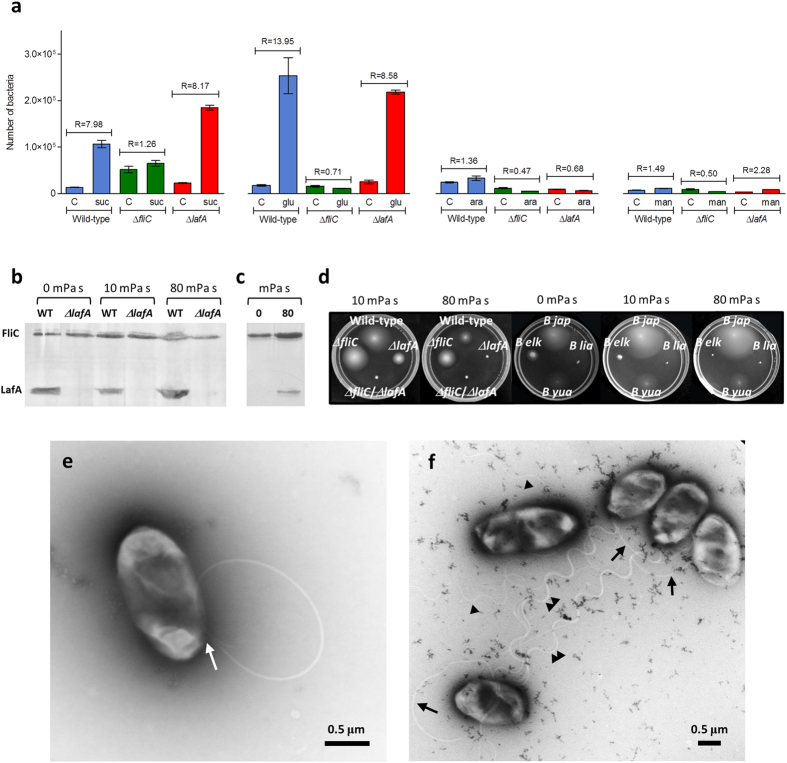
Different contributions of each flagellar system to chemotaxis and motility. (**a**) Chemotaxis towards succinate (*suc*), glutamate (*glu*), arabinose (*ara*) and mannitol (*man*). Above the pair of bars corresponding to a combination of chemoattractant and control (*C*), the chemotaxis ratio (*R*) is indicated, which is the ratio of colony-forming units inside capillary with chemoattractant to colony-forming units inside capillary without chemoattractant. The results are the average ± confidence interval (95%). (**b,c**) Production of subpolar (FliC) and lateral (LafA) flagellins in Götz medium with mannitol as the sole carbon source at the indicated viscosities. (**b**) Wild-type (WT) and Δ*lafA* in soft agar. (**c**) WT in liquid medium. (**d**) Effects of viscosity on swimming in Götz soft agar with mannitol as the sole carbon source in *B. diazoefficiens* wild-type, Δ*lafA*, Δ*fliC* and Δ*lafA*/Δ*fliC*, as well as in *B. japonicum* USDA 6 (*B jap*), *B. liaoningense* 2281 (*B lia*), *B. elkanii* USDA 76 (*B elk*) and *B. yuanmingense* CCBAU 10071 (*B yua*). (**e,f**) Transmission electron micrographs of *B. diazoefficiens* cells obtained from Götz soft agar with mannitol as the sole carbon source, supplemented with PVP K-90 to yield a viscosity of 80 mPa s. (**e**) Δ*lafA* showing its subpolar flagellum attached to the cell body (*arrow*). The flagellum describes a curve and passes over the cell, finishing near the insertion point. (**f**) Five wild-type cells, showing subpolar (*arrows*) and lateral (*arrowheads*) flagella attached to the cell bodies. Some lateral flagella are associated in bundles of more than two filaments (*double arrowheads*) while others are single (*single arrowheads*). Scale bar: 0.5 μm.

In agreement with data in USDA 110 28, succinate and glutamate were strong chemoattractants for the wild-type strain, whereas mannitol and arabinose were weak. Furthermore, Δ*lafA* behaved similarly to the wild-type, while Δ*fliC* did not respond to any of the four chemoattractants that were assayed (Fig. 7a). Because all of strains entered the capillaries without chemoattractant at similar rates, the low chemotaxis ratio (*R*) values of Δ*fliC* cannot be attributed solely to its slower swimming speed. Together, these results indicate that the subpolar flagellum is enough for a chemotactic response in this assay.

### Soft agar induces the lateral flagellum, which is required for motility with increasing viscosity

We previously observed that swimming of Δ*lafA* in soft agar (0.3% w/v) with mannitol as the sole carbon source was slower than the wild-type and similar to Δ*fliC*^18^. Because LafA are not produced in liquid medium with mannitol as the sole carbon source^17^,^18^,^19^, we wondered whether the contact with the agar mesh might induce LafA production. To evaluate this possibility, we extracted the extracellular proteins of bacteria cultured in Götz soft agar (0.3% w/v) with mannitol as the sole carbon source, and observed that LafA was produced in this condition (Fig. 7b). We also tested whether an increase in viscosity may induce the production of LafA^15^, and observed that this protein was produced both in liquid and soft agar at 10 and 80 mPa s (Fig. 7b,c). Moreover, a viscosity of 10 mPa s in soft agar reduced the motility of Δ*lafA* but not that of Δ*fliC*, while at 80 mPa s, Δ*lafA* was completely halted, similarly to the Δ*fliC*/Δ*lafA* non-motile mutant (Fig. 7d). Because in all these conditions FliC was synthesised at similar levels (Fig. 7b) and the subpolar flagella were attached to the cells (Fig. 7e,f), the paralysis of Δ*lafA* is not attributable to suppression of subpolar flagella production (additional examples of Δ*lafA* cells in soft agar at 80 mPa s are provided in Supplemetary Fig S3). By contrast, the wild-type and Δ*fliC* strains remained motile, indicating that the lateral flagella always were functional. We also evaluated whether this behaviour is reproduced in soybean-nodulating *Bradyrhizobium* wild-type strains with natural differences in their flagellar systems (Fig. 1b). Thus, we observed that *B. japonicum* USDA 6 and *B. yuanmingense* CCBAU 10071 were not affected by viscosity in soft agar, *B. elkanii* USDA 76 was affected at 10 mPa s and unable to move at 80 mPa s, and *B. liaoningense* 2281 did not move at all (Fig. 7d), in agreement with the lack of *flhB* and *fliF* in strains CCBAU 05525 and CCBAU 83689.

## Discussion

Biological systems may be considered dynamic entities whose performances are dictated by their internal complex organization in interaction with the environment^30^,^31^. Thus, performance was referred to as the ability to execute an ecologically relevant task^32^, in which sense flagella-driven swimming motility is among the most important and energy-consuming performance of bacterial cells. *B. diazoefficiens* is intriguing because, by difference with most of the species with dual flagellar systems, it may express its two flagellar systems simultaneously in liquid medium, thus raising the question about their ecological functions and adaptive values. Our cumulative 6–8 min of tracking of wild-type, Δ*lafA* and Δ*fliC* indicated that the swimming behaviour of mutant cells that were endowed with one or the other flagellar system had distinct features in agreement with each system’s properties. In addition, the instability of Δ*fliC* trajectories suggests that lateral flagella do not form stable bundles. However, when both flagellar systems were present together, there was a high frequency of circular trajectory shapes that, according to statistical analysis of *TRAIN* values, was not due to the additive effects of each flagellar system, but an interaction between them. Thus, swimming performance of wild-type cells is an emergent property that integrates the functions of both flagellar systems to be used together for the single task of swimming^33^. Such an emergent property could not be observed before in other species with dual flagellar systems because most of them do not express their lateral flagella in planktonic cells.

Swimming cells were tracked near the glass surfaces, as indicated by tethered cells often found rotating around a given point in the same focal plane. Therefore, at least part of the trajectories should finish when cells attach to the glass surface^34^. Hence, it might be useful to compare the trajectory durations and the attachment to glass among the mutants and the wild-type to better understand the relationship between swimming and surface interactions. On the one hand, duration of Δ*fliC* trajectories was substantially higher than those of the other strains and its attachment to glass surface was low and indistinguishable from Δ*fliC*/Δ*lafA*, indicating that, by difference with model systems such as *Vibrio* and *Aeromonas*^15^, lateral flagella of *B. diazoefficiens* are poor adhesins. On the other hand, the duration of linear trajectories of Δ*lafA* was shortest, and this strain had three-fold higher attachment to the glass surface than the wild-type. Because the swimming speeds of the wild-type and Δ*lafA* were similar, their difference in attachment to glass might be related with an effect of lateral flagella^35^. Circular trajectories occur mostly when bacteria are swimming persistently near a surface, whereas opposite torques are produced on the flagellum and the cell body^36^,^37^. Hence, the hydrodynamic forces experienced by the cell body against the glass surface, such as friction and drag^34^,^38^, should differ if the cell body is surrounded by lateral flagella or not. Thus, assuming that lateral flagella are poor adhesins, they may impair premature docking and attachment of the wild-type. In this way, lateral flagella might favour the persistent swimming near the glass surface as suggested by wild-type trajectory shapes, while the subpolar flagella might be responsible for propulsion and direction, as suggested by wild-type swimming speed, predominant changes in direction by the RR and RRF patterns, and chemotaxis response. The role of lateral flagella in propulsion seems restricted to viscous environments.

It is interesting that LafA was induced in soft agar (0.3% w/v) without the addition of a viscous agent, by difference to *V. parahaemolyticus* and *Plesiomonas shigelloides*, which require harder agar for lateral flagellar genes induction^39^,^40^,^41^. The agar concentration often defines whether bacterial cells swim in the water-filled spaces of the agar (0.2–0.4% w/v) or swarm on the surface of the agar (more than 0.5% w/v) aided by surfactants secreted by the bacterial cells^42^. In the above-mentioned species, as well as in most of the species with dual flagellar systems, the mechanical signals for lateral flagella induction are the contact with surfaces and the viscosity. Our results suggest that in the case of *B. diazoefficiens* the presence of obstacles (e.g. the agar mesh) may be a mechanical signal for lateral flagella induction, which seems relevant in the soil.

The different roles of lateral flagella in adhesion and swimming with respect to model systems might be related with the different activities of lateral flagella in swarming. While in *Vibrio* spp. and *Aeromonas* spp. lateral flagella are used for swarming on surfaces^15^,^16^,^42^, in *B. diazoefficiens* swarming is not as evident and the requirement of these flagella for swarming is not so specific^19^. Therefore, in this bacterial species the role of swarming on surfaces might be replaced by the sustained swimming near surfaces, which might explain why the lateral flagella are expressed in planktonic state, and work in interaction with subpolar flagella. In addition, swarming is a social movement while swimming is restricted to individual cells^16^, whereby sustained swimming near surfaces should not require the association of a high number of cells. Whether this behaviour is adapted to the size of *B. diazoefficiens* soil populations or to the special properties of soil inner surfaces is a matter of future research.

**Figure 8:**
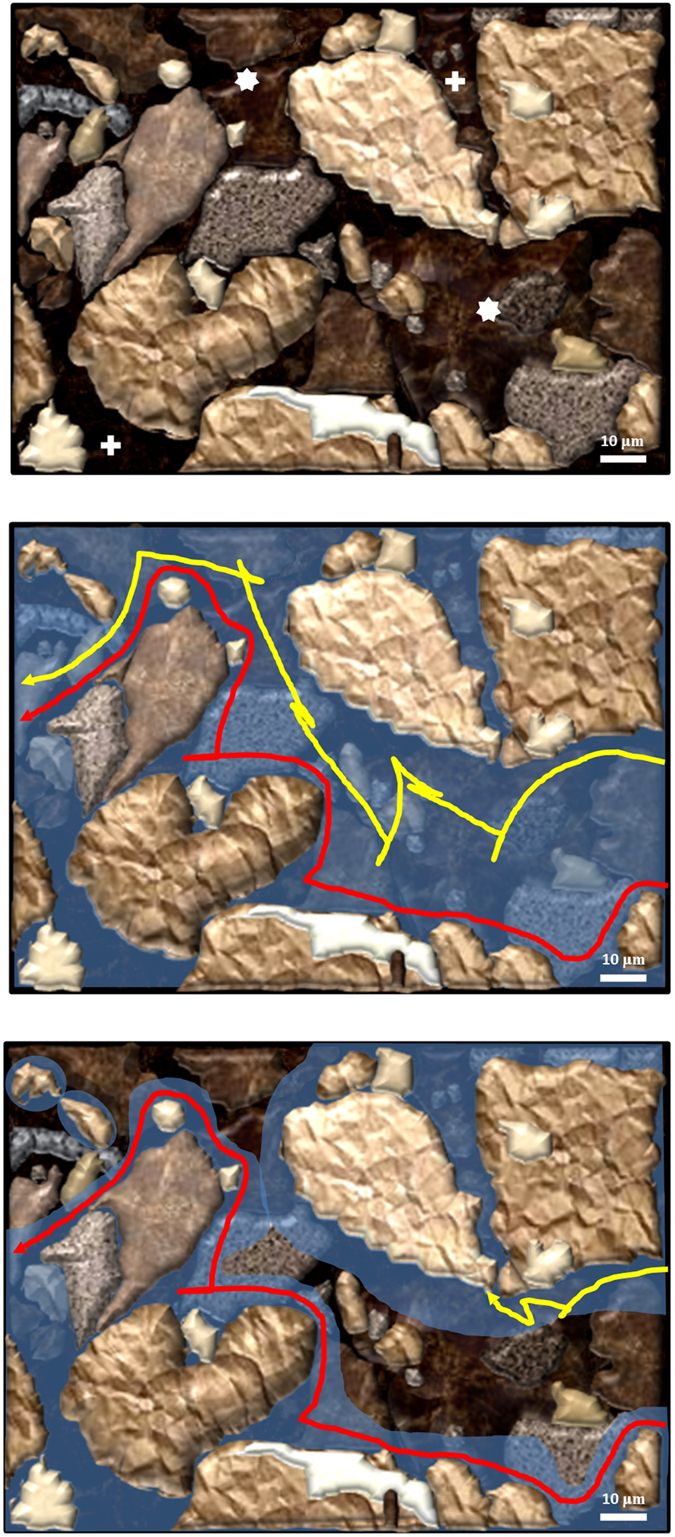
Sketch of the different strategies of *Bradyrhizobium* dispersion into the soil. *Top panel*: a magnification of a dry silt loam soil is represented, with a macropore (*asterisks*) and two micropores (*crosses*). *Middle panel*: the same soil saturated with water. The trajectories of two bacteria are shown: one bacterium swims near the surfaces (*red*) and the other swims in the bulk liquid (*yellow*). *Bottom panel*: part of the gravitational water was lost, and the water remaining in the macropore forms layers on the walls; now the bacterium represented by the *yellow* trajectory cannot swim freely and hits one of the walls. Scale bar: 10 μm.

This dissection that was made with deletional mutants in the same genomic background may allow us to envisage the role of each flagellar system in the soil, which is an opaque matrix where the live observation of swimming bacteria is a technical challenge not yet resolved. The soil pores (Fig. 8a) constitute an environment with steep changes in water content, water potential, viscosity and chemical composition, leading to heterogeneities both in space and time, which depend on pore diameters and water inputs to the soil. At water saturation all soil channels are filled with water and *Bradyrhizobium* bacteria can swim freely inside macropores, either near the channel surfaces or in the bulk liquid (Fig. 8b). When the gravitational water begins to drain, the macropores retain only a layer of water. Under these conditions, the expression of dual flagellar systems might allow persistent swimming at a proper distance from the surface, oriented to chemoattractant sources in a strategy of robust exploration. Meanwhile, when the cells devoid of lateral flagella are forced to swim near the surfaces because of the loss of gravitational water, they would rapidly hit the inner walls of the soil channels leading to cell attachment and microcolony formation (Fig. 8c). Therefore, cells with uninduced lateral flagellar systems should take advantage of short events of water saturation for rapid dispersal and further attachment to and colonization of surfaces^43^.

These two strategies of dispersal might coexist in soils populated by *Bradyrhizobium* species with single or dual flagellar systems. *B. diazoefficiens*, *B. japonicum* and *B. elkanii* are extensively present in soils of the soybean areas of Brazil, China, India and Paraguay^44^,^45^,^46^,^47^. Likewise, in the closely related *R. palustris*, strains with dual or single flagellar systems were isolated from the same 0.5-g freshwater sediment sample^48^. Finally, the soybean-nodulating *B. liaoningense* that lacks *flhB* and *fliF* is non-motile in soft agar. Therefore, it is tempting to speculate that a diversity of swimming behaviours may constitute a multistable adaptation to such complex and structured environments. The fact that the secondary flagellar system appears repeatedly in different lineages and the failure of the search for evidence of lateral flagella horizontal gene transfer both in *Bradyrhizobium* and *Rhodopseudomonas* support the hypothesis that an ancestor of these bacterial genera had both flagellar systems, the lateral one being lost in some descendant lineages^14^. Gene loss might be advantageous even in free-living microorganisms if the lost function is an expensive one that can be provided by other members of the community (“helpers”), up to the point that the benefit that is obtained by the members sparing the function is compensated for by the cost that would result if the fraction of helpers were absent^49^. Although, to our knowledge, cooperation among strains for flagella-driven motility was not yet reported, it is known that at the rim of expanding colonies of *Bacillus subtilis*, flagellated cells associate with non-flagellated ones^50^,^51^, and in the social motility of *Myxococcus xanthus*, the cooperation among natural isolates with high and low proficiency for motility was documented^52^. Moreover, strains with superior swimming and swarming motilities, as well as de-repression in flagella production, were easily obtained by artificial evolution in the laboratory^17^,^19^,^53^, indicating that the motility and regulation of flagellar expression are not necessarily fitted to their maxima, but to levels that are optimal for the plasticity for the choice of motility and colonization strategies in the environment. Taken together, these results suggest that the integration of flagellar function in interaction with the environment may occur at both the cell and the community levels as a general adaptation to structured complex habitats.

## Methods

### Strains and growth conditions

*B. diazoefficiens* LP 3004, LP 6865, LP 5843, and LP 6543 were described elsewhere^18^. LP 3004 is a wild-type derivative from USDA 110^T^ with spontaneous resistance to streptomycin. LP 6865 (Δ*lafA*) is a derivative from LP 3004 in which 97.8% of the coding sequences of *lafA12* (bases 7561160–7563387) were replaced with a streptomycin-spectinomycin-resistance cassette. LP 5843 (Δ*fliC*) is a derivative from LP 3004 in which 75.6% of the *fliC1234*-coding sequences (bases 6410728–6418519) were replaced by a kanamycin-resistance cassette. LP 6543 (Δ*fliC1234*/Δ*lafA12*) is a non-motile derivative from LP 6865 with the same mutation of LP 5843. *B. japonicum* USDA 6^T^, *B. elkanii* USDA 76^T^, *B. liaoningense* 2281^T^ and *B. yuanmingense* CCBAU 10071^T^ were kindly provided by Dr. Esperanza Martínez-Romero, UNAM, Mexico. Unless specified, strains were grown in liquid AG medium^54^ at 30 °C with rotary shaking at 180 rpm.

### Bioinformatic analyses

Sequences of FlhA, FlhB, FliF, FliG and FliP from each organism were recovered from http://img.jgi.doe.gov or http://www.ncbi.nlm.nih.gov. These sequences were concatenated by hand, and multiple sequence alignments were performed with Clustal W^55^ and MEGA6 software^56^ using the neighbour-joining method with 1,000 bootstrap replicates.

### Characterization of swimming

Bacteria were harvested at an optical density at 500 nm (OD_500_) of 1.0 and diluted in Götz minimal medium without a carbon source^57^. The cells were left at 30 °C without agitation for 24 h and then observed and recorded as described^27^. Briefly, swimming cells of each strain were tracked at 30 frames s^−1^ in videos of 5–6 s of duration with a phase contrast Axioscope 2 Carl Zeiss light microscope (Jena, Germany) that was connected to a Sony Exwave HD video camera (Tokyo, Japan). Computer-assisted motion analysis of the digitized images was performed with CellTrak (version 1.5, Motion Analysis Corporation, Santa Rosa, CA, USA). From these videos, we determined the coordinate position of each cell in the *x*-*y* space.

### Attachment to glass slides

Bacterial suspensions that were incubated as described for swimming observations were diluted 1:10 in Götz minimal medium without a carbon source^57^, and 200 μl was inoculated onto the surface of each glass slide. In parallel, the number of bacteria in the suspension was counted in a Neubauer chamber. The slides were incubated for 1 h at room temperature without agitation and then subjected to four washes of 1 min at 100 rpm with 15 ml of distilled water. Then, the slides were fixed, stained with crystal violet, and washed with water, and the stained cells were counted under the microscope at 400× magnification. Ten fields per slide were counted and summed, and the average of these sums in three slides was calculated. Attachment was expressed as these averages divided by the number of cells inoculated, according to counts in the Neubauer chamber.

### Chemotaxis and swimming in soft agar plates

These experiments were carried out with cells prepared as described above for swimming and attachment experiments, and then suspended in Götz minimal medium with 10 mM tested carbon source. The chemical-in-capillary assays of Adler with some modifications were performed as described with four capillaries per determination^27^. The plate assays were performed with 0.3% w/v agar as described^17^. To increase the medium viscosity, polyvinylpyrrolidone (PVP) K-90 was added at 2% w/v or 5% w/v, resulting in viscosities of 10 or 80 mPa s, respectively, according to previous measurements^58^.

### Flagellin separation and analysis

Bacteria were grown in Götz medium with mannitol as the sole carbon source, either in liquid or in soft agar (0.3% w/v). Bacteria were collected from liquid cultures when their OD (500 nm) reached 1.0. To collect bacteria from soft agar plates, 8-day old 10–110 bacterial colonies (according to their diameters, which ranged 0.3–3.5 cm) were transferred to one 50-ml centrifuge tube, and one volume of sterile distilled water was added. Afterwards, the tubes were kept for 15 min on ice, vortexed for 5 min, and centrifuged at 10,000 × g for 30 min at 4 °C. The supernatants, or the liquid cultures mentioned above, constituted the starting materials for flagellin enrichment. These fractions were incubated with 1.3% w/v polyethylene glycol 6000 and 166 mM NaCl for 2 h at 4 °C. This suspension was centrifuged at 11,000 × g for 40 min at 4 °C, and the pellet was resuspended in Laemmli buffer^59^. For analysis, the samples were boiled for 10 min and then separated by sodium dodecyl sulfate polyacrylamide (12.5% w/v) gel electrophoresis^59^. Polypeptide bands were revealed by silver-staining.

### Transmission electron microscopy

Bacteria were obtained either from liquid cultures or from beneath the surface of swimming agar (0.3%) plates. The observations were carried out at the Microscopy Service, Faculty of Veterinary Sciences, UNLP as described^17^. Briefly, aliquots were transferred to a grid covered with a colodion film over which carbon was previously layered through vacuum evaporation. After 30 seconds, excess liquid was removed with filter paper and the sample was stained with 2% potassium phosphotungstate (pH 5.2; 2% w/v KOH). The microscope employed was a JEM 1200 EX (JEOL, Japan Electron Optics Laboratory Co., Ltd.).

### Statistics

To appreciate the extent of variation of *TRAIN* values within samples, *TRAIN* was expressed as average ± SD. All other values, when presented to appreciate the differences between experimental means, are presented as mean ± confidence interval. Confidence intervals were obtained by multiplying the standard mean errors by the Student’s-*t* with α = 0.05. Factorial analysis was carried out with sets of 20 trajectories of each strain chosen at random and checked for normality with the Anderson-Darling test with α = 0.05. Sets that passed the Anderson-Darling test were analyzed in a 2 × 2 factorial design for the effects of subpolar flagella, lateral flagella and their interaction on the *TRAIN* values. Because the non-motile double mutant Δ*fliC*/Δ*lafA* was immobile under the microscope and therefore could not be tracked by the software, we assigned it *TRAIN* = 0 with one degree of freedom. We believe that this assumption is validated by the average *TRAIN* = 0.05 of Δ*fliC* diffusive trajectories, because average *TRAIN* of Δ*fliC*/Δ*lafA* should be necessarily lower.

## Additional Information

**How to cite this article**: Quelas, J. I. *et al*. Swimming performance of *Bradyrhizobium diazoefficiens* is an emergent property of its two flagellar systems. *Sci. Rep*. **6**, 23841; doi: 10.1038/srep23841 (2016).

## Supplementary Material

Supplementary Information
