# Supplementary material for: Swimming performance of Bradyrhizobium diazoefficiens is an emergent property of its two flagellar systems
Source: Sci Rep. 2016 Apr 7;6:23841. doi: 10.1038/srep23841 (PMC4823718; doi:10.1038/srep23841)
Supplement: Supplementary Information [file srep23841-s1.pdf]

Swimming performance of *Bradyrhizobium diazoefficiens* is an emergent property of its two flagellar systems

Supplementary Information

J. Ignacio Quelas[a], M. Julia Althabegoiti[a], Celia Jimenez-Sanchez[a,b], Augusto A. Melgarejo[c], Verónica I. Marconi[d], Elías J. Mongiardini[a], Sebastián A. Trejo[e,f], Florencia Mengucci[a], José-Julio Ortega-Calvo[b], Aníbal R. Lodeiro[a*]

[a]Instituto de Biotecnología y Biología Molecular (IBBM)-Facultad de Ciencias Exactas, UNLP-CONICET (Argentina), [b]Instituto de Recursos Naturales y Agrobiología de Sevilla (IRNAS)-CSIC (Spain), [c]Departamento de Ciencias Básicas, Facultad de Ingeniería, UNLP (Argentina), [d]Facultad de Matemáticas, Astronomía y Física (FAMAF)-UNC e IFEG-CONICET (Argentina), [e]Servei de Proteòmica i Biologia Estructural, Universitat Autònoma de Barcelona, Bellaterra (Spain). [f]Present address: Instituto Multidisciplinario de Biología Celular (IMBICE), CONICET (Argentina).

Matrix-assisted laser desorption ionization time-of-flight (MALDI-TOF) analysis of subpolar and lateral flagellins. Polypeptide samples were taken from single bands of denaturing polyacrylamide gel (SDS-PAGE) separations of subpolar and lateral flagellin subunits. These bands were trypsin-digested and analyzed essentially as described[60]. For MALDI-TOF target preparation, each sample was spotted onto a MALDI target plate (MTP) 384 GroundSteel plate (Bruker Daltonik, Bremen, Germany) using a standard dried droplet method. Profile analyses were performed on an UltrafleXtreme MALDI TOF/TOF spectrometer with FlexControl 3.4 (Bruker Daltonik, Bremen, Germany) data acquisition software. Mass spectra were acquired in reflector mode geometry using the following settings: 600-5000 Da mass range, positive mode, and ion suppression up to 800 m/z. Mass calibration was performed using the Peptide Calibration Standard (Bruker Daltonik, Bremen, Germany).

LafA1 and LafA2 (Bll6866 and Bll6865, respectively) were unambiguously identified from the SDS-PAGE low-molecular-weight band by peptide mass fingerprint MALDI TOF mass spectrometry (MS) using the MASCOT tools search. Similarly, only some FliC proteins were identified from the SDS-PAGE high-molecular-weight band. A further analysis that was carried out by sequencing MALDI TOF-TOF MS (Supplementary Table S1) allowed a clear identification of specific tryptic peptides from each flagellin (FliC1, FliC2, FliC3 and FliC4). The obtained chromatograms are shown in Supplementary Fig. S1, and the specific peptides sequenced are resumed in Supplementary Table S2. Therefore, all of the polypeptides that were encoded in bll6865-bll6866 and bll5843-bll5846 were identified in our *B. diazoefficiens* flagellin samples.

Table S1. Peptides identified in theoretical tryptic digestion of flagellins*.

	Meas M/z	Calc MH+	Dev.(Da)	Range	Sequence
**637373431 NP_772483 [B. japonicum USDA 110: NC_004463]**					
peak 40	2454,40	2454,37	0.034	741 - 763	QSIAVSALSLANQSQQSVLQLLR
peak 17	1681,95	1681,88	0.070	93 - 108	SIANQALQTTVGYSTK
peak 38	2336,20	2336,16	0.041	36 - 56	KVNTALDNPTNFFTAQGLDNR
peak 29	2208,10	2208,07	0.035	37 - 56	VNTALDNPTNFFTAQGLDNR
peak 11	1616,92	1616,86	0.061	689 - 704	SEGSALGSNLSIVQVR
peak 28	2102,15	2102,11	0.038	12 - 30	QNLLSLQSTADLLATTQER
peak 46	2632,30	2632,29	0.008	501 - 526	TLTFTSFNGGTPVNVTFGDGTNGTVK
peak 49	2734,43	2734,39	0.035	347 - 373	STTAGSLGTLVQDGSTLNIDGHTITFK
peak 63	3746,28	3746,01	0.271	1 - 35	MSNIVLSASVRQNLLSLQSTADLLATTQERLSTGK
**637373432 NP_772484 [B. japonicum USDA 110: NC_004463]**					
peak 35	2311,14	2311,10	0.041	36 - 56	SVNSALDNPTNFFTAQSLDNR
peak 27	2087,15	2087,11	0.045	12 - 30	QNLLSLQSTADLLATTQNR
peak 40	2454,40	2454,37	0.034	735 - 757	QSIAVSALSLANQSQQSVLQLLR
peak 47	2647,39	2647,36	0.029	337 - 363	TTSAASLGATIADGSTLNVDGHVITFK
peak 32	2273,20	2273,17	0.038	628 - 648	LVFDETGKSSLNITGVTYNSK
peak 17	1681,95	1681,88	0.070	93 - 108	SIANQALQTTVGYSTK
peak 14	1646,95	1646,87	0.078	683 - 698	SEASSLGSNLSVVQIR
peak 5	1359,81	1359,76	0.046	670 - 682	VLTNLNAASSTLR
peak 22	1817,05	1816,95	0.103	318 - 336	ALGLTTSTGAGNATVNVNR
peak 50	2915,61	2915,57	0.046	268 - 300	TVSISSGAATIAVSASQPGAAVSTAAAGAVTLK
peak 42	2573,40	2573,39	0.007	12 - 35	QNLLSLQSTADLLATTQNRLSTGK
peak 1	932,56	932,51	0.056	521 - 528	TLDQLNTK
peak 7	1383,73	1383,71	0.015	636 - 648	SSLNITGVTYNSK
**637373433 NP_772485 [B. japonicum USDA 110: NC_004463]**					
peak 35	2311,14	2311,10	0.041	36 - 56	SVNSALDNPTNFFTAQSLDNR
peak 21	1785,04	1784,96	0.077	318 - 336	ALGLTTAVGGGNATVNVNR
peak 40	2454,40	2454,37	0.034	735 - 757	QSIAVSALSLANQSQQSVLQLLR
peak 47	2647,39	2647,36	0.029	337 - 363	TTSAASLGATIADGSTLNVDGHVITFK
peak 32	2273,20	2273,17	0.038	628 - 648	LVFDETGKSNLSITGVTYNSK
peak 17	1681,95	1681,88	0.070	93 - 108	SIANQALQTTVGYSTK
peak 25	2060,14	2060,10	0.042	12 - 30	QNLLSLQSTADLLATTQSR
peak 15	1660,97	1660,89	0.082	683 - 698	SEASSLGSNLTIVQVR
peak 43	2576,29	2576,26	0.025	495 - 520	TLTFASFNGGTAVNVTFGDGTNGTVK
peak 34	2293,16	2293,18	-0.022	683 - 703	SEASSLGSNLTIVQVRQDFNK
peak 6	1375,80	1375,75	0.050	670 - 682	VLTNLNSASSTLR
peak 57	3374,92	3374,79	0.128	649 - 682	GLGLAALTGGVDFIDNAATNKVLTNLNSASSTLR
peak 62	3492,98	3492,79	0.185	448 - 484	LSTGVNADLSVTGTGNALNVLGLAGNTGTSTAFTAAR
peak 23	2018,11	2018,06	0.052	649 - 669	GLGLAALTGGVDFIDNAATNK
peak 52	3038,66	3038,61	0.050	57 - 86	ASDINNLLDGIANGVQVLQAANTGITSLQK
peak 36	2325,34	2325,29	0.050	313 - 336	ADLLKALGLTTAVGGGNATVNVNR
peak 60	3428,99	3428,83	0.153	301 - 336	SSTGADLSVTGKADLLKALGLTTAVGGGNATVNVNR
peak 9	1563,89	1563,80	0.088	109 - 124	SNVSTTISGATAADLR
peak 50	2915,61	2915,57	0.046	268 - 300	TVSISSGAATIAVSASQPGAAVSTAAAGAVTLK
peak 61	3433,88	3433,72	0.153	485 - 520	TSGVGGITGKTLTFASFNGGTAVNVTFGDGTNGTVK
peak 41	2546,25	2546,38	0.127	12 - 35	QNLLSLQSTADLLATTQSRLSTGK
peak 1	932,56	932,51	0.056	521 - 528	TLDQLNTK
peak 7	1383,73	1383,71	0.015	636 - 648	SNLSITGVTYNSK
peak 63	3746,28	3745,92	0.362	409 - 447	AIDLATGVQTATINANGTATLATATGQTNSSINASGQLK

**637373434 NP_772486 [B. japonicum USDA 110: NC_004463]**					
peak 35	2311,14	2311,10	0.041	36 - 56	SVNSALDNPTNFFTAQSLDNR
peak 21	1785,04	1784,96	0.077	318 - 336	ALGLTTAVGGGNATVNVNR
peak 31	2265,18	2265,15	0.034	683 - 703	SEASSLGSNLSVVQVRQDFNK
peak 27	2087,15	2087,11	0.045	12 - 30	QNLLSLQSTADLLATTQNR
peak 40	2454,40	2454,37	0.034	735 - 757	QSIAVSALSLANQSQQSVLQLLR
peak 47	2647,39	2647,36	0.029	337 - 363	TTSAASLGATIADGSTLNVDGHVITFK
peak 13	1632,94	1632,86	0.081	683 - 698	SEASSLGSNLSVVQVR
peak 4	1345,79	1345,74	0.049	670 - 682	VLSNLNAASSTLR
peak 32	2273,20	2273,17	0.038	628 - 648	LVFDETGKSNLSITGVTYNSK
peak 17	1681,95	1681,88	0.070	93 - 108	SIANQALQTTVGYSTK
peak 43	2576,29	2576,26	0.025	495 - 520	TLTFASFNGGTAVNVTFGDGTNGTVK
peak 24	2048,12	2048,07	0.054	649 - 669	GLGLAALTSGVDFIDNAATNK
peak 57	3374,92	3374,79	0.128	649 - 682	GLGLAALTSGVDFIDNAATNKVLSNLNAASSTLR
peak 62	3492,98	3492,79	0.185	448 - 484	LSTGVNADLSVTGTGNALNVLGLAGNTGTSTAFTAAR
peak 52	3038,66	3038,61	0.050	57 - 86	ASDINNLLDGIANGVQVLQAANTGITSLQK
peak 36	2325,34	2325,29	0.050	313 - 336	ADLLKALGLTTAVGGGNATVNVNR
peak 8	1533,86	1533,79	0.076	109 - 124	SNVSATISGATAADLR
peak 60	3428,99	3428,83	0.153	301 - 336	SSTGADLSVTGKADLLKALGLTTAVGGGNATVNVNR
peak 51	2959,61	2959,58	0.028	670 - 698	VLSNLNAASSTLRSEASSLGSNLSVVQVR
peak 50	2915,61	2915,57	0.046	268 - 300	TVSISSGAATIAVSASQPGAAVSTAAAGAVTLK
peak 61	3433,88	3433,72	0.153	485 - 520	TSGVGGITGKTLTFASFNGGTAVNVTFGDGTNGTVK
peak 42	2573,40	2573,39	0.007	12 - 35	QNLLSLQSTADLLATTQNRLSTGK
peak 55	3190,69	3190,62	0.072	704 - 734	SLINVLQTGSSNLTLADTNTEAANSQALSTR
peak 1	932,56	932,51	0.056	521 - 528	TLDQLNTK
peak 7	1383,73	1383,71	0.015	636 - 648	SNLSITGVTYNSK
peak 64	3746,28	3745,92	0.362	409 - 447	AIDLATGVQTATINANGTATLATATGQTNSSINASGQLK
peak 66	3824,33	3824,00	0.328	87 - 124	LIDSAKSIANQALQTTVGYSTKSNVSATISGATAADLR
peak 65	3823,25	3822,91	0.343	699 - 734	QDFNKSLINVLQTGSSNLTLADTNTEAANSQALSTR
peak 63	3592,05	3591,87	0.179	670 - 703	VLSNLNAASSTLRSEASSLGSNLSVVQVRQDFNK

*All matches between spectrum mass peak and theoretical tryptic digest peptides of each flagellin are shown. Grey rows: specific peptides from each flagellin. Specific peptide corresponds to sequences present only in one flagellin but not in the others.

637373431 NP_772483:

Peak 28

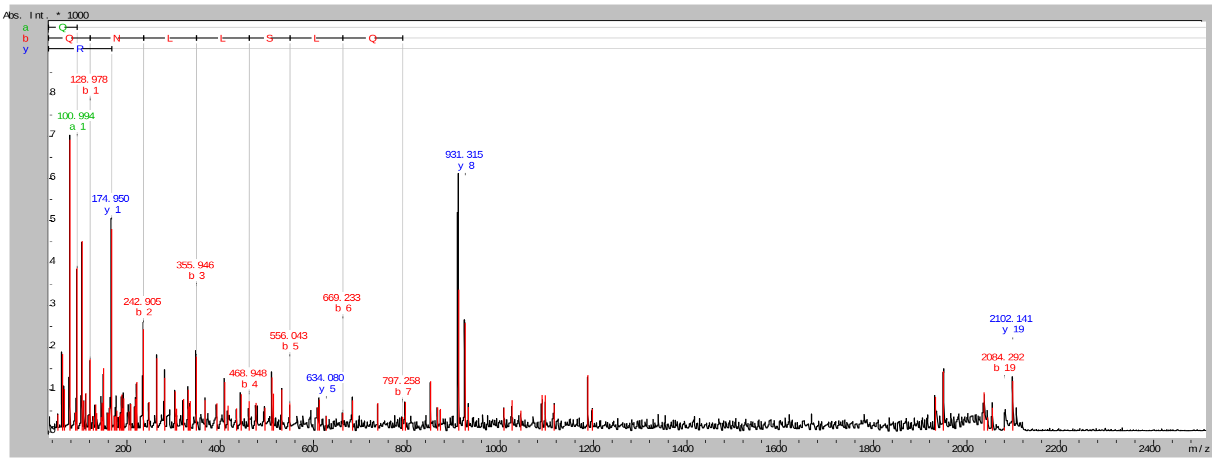

Peak 38

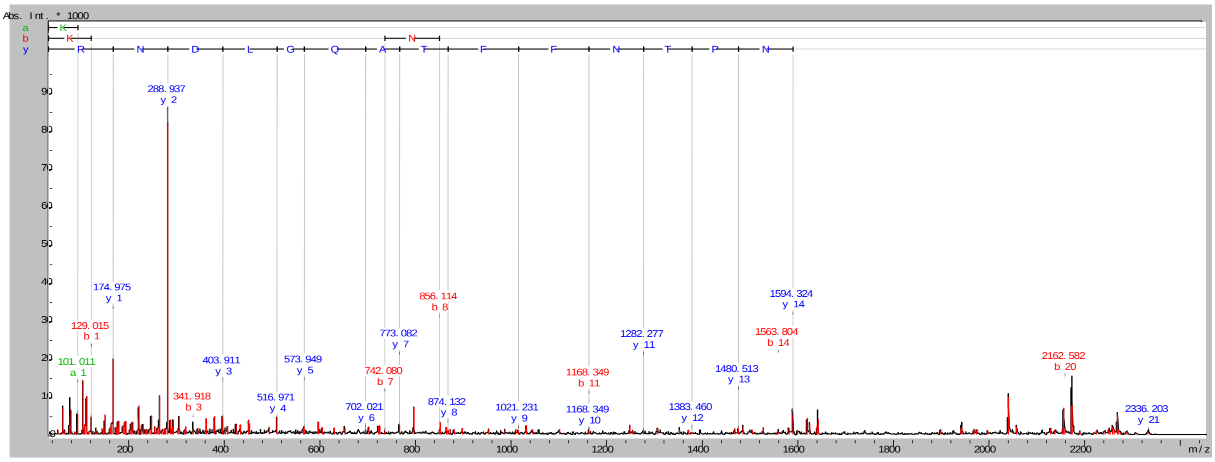

637373432 NP_772484:

Peak 5

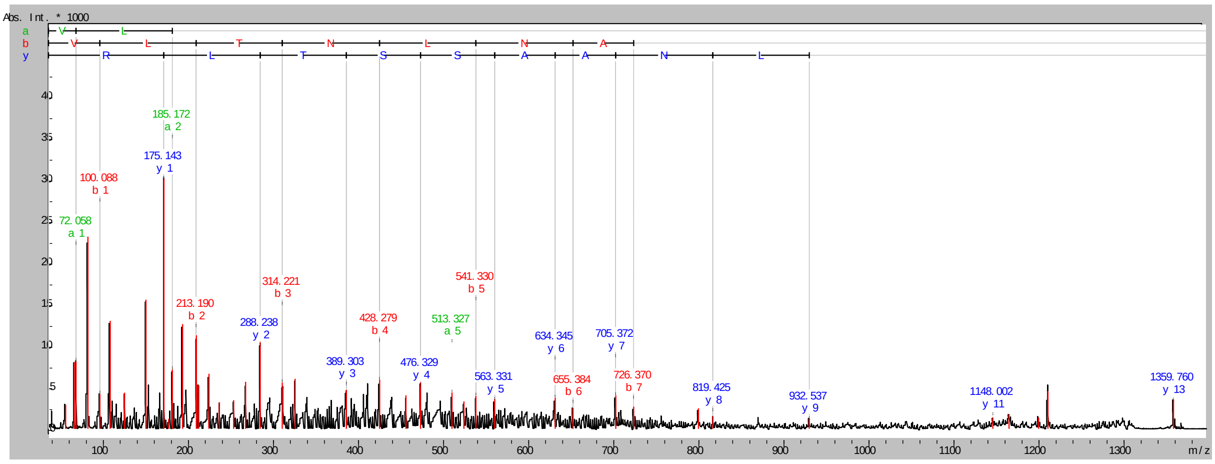

Figure S1

Peak 14

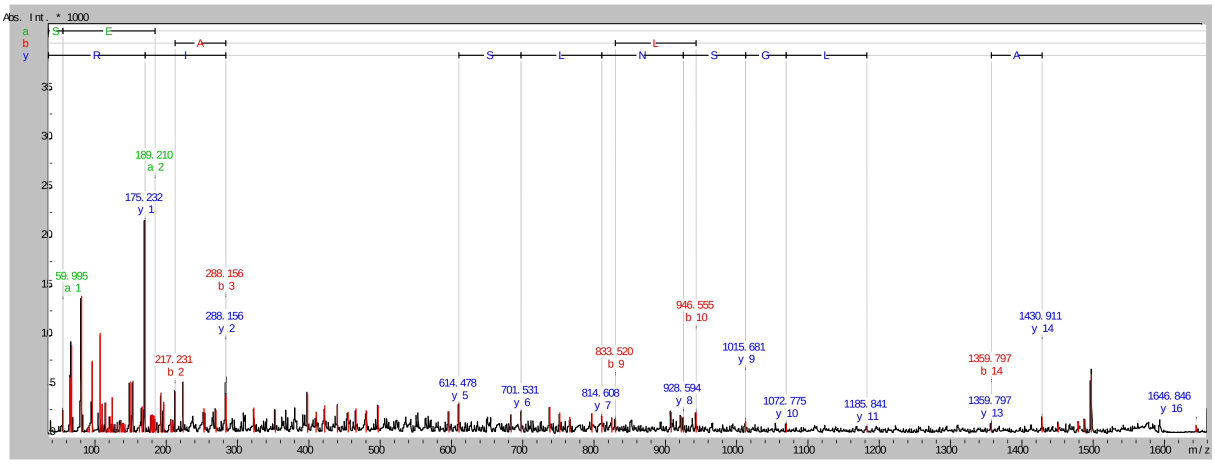

Peak 22

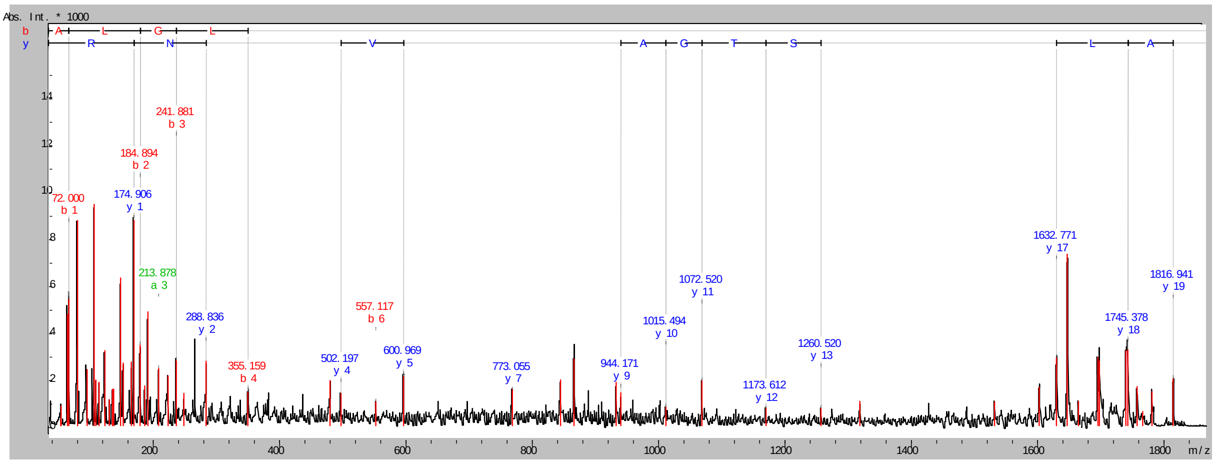

637373433 NP_772485:

Peak 15

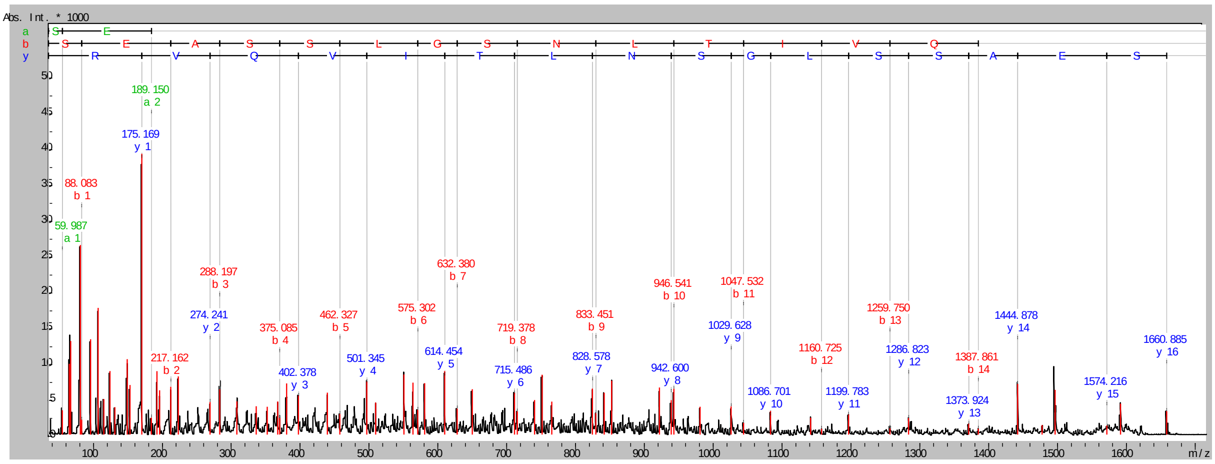

Figure S1 (cont.)

Peak 25

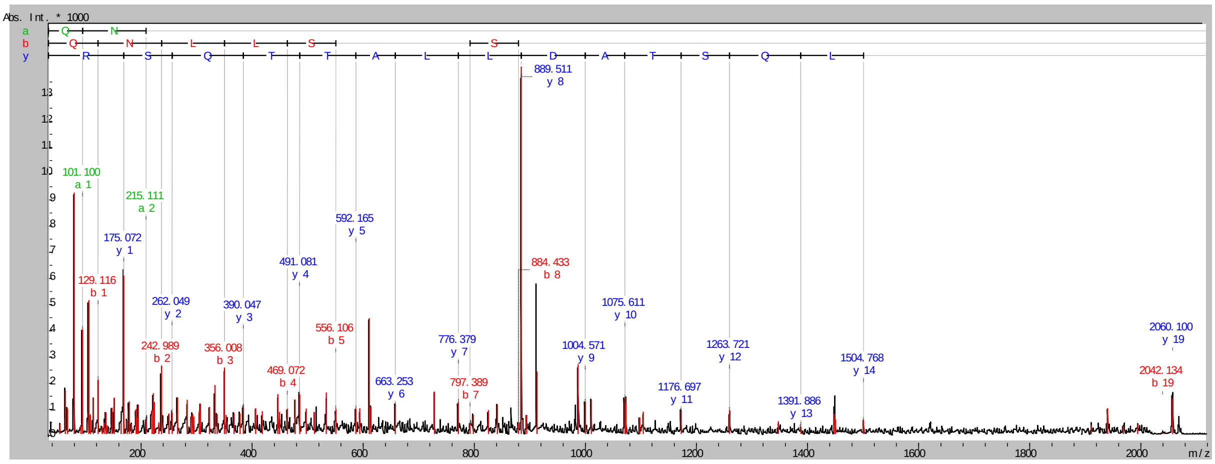

Peak 35

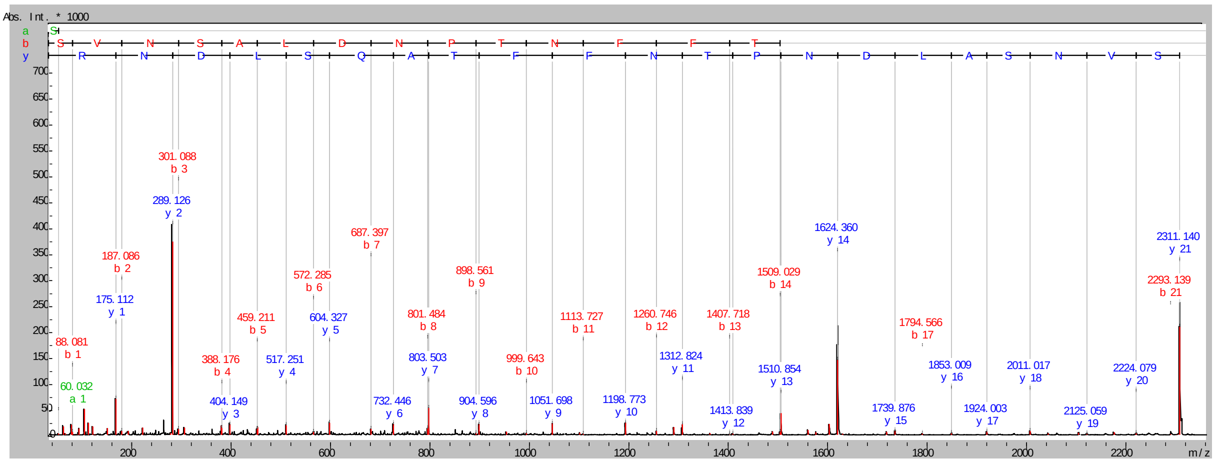

637373434 NP_772486:

Peak 4

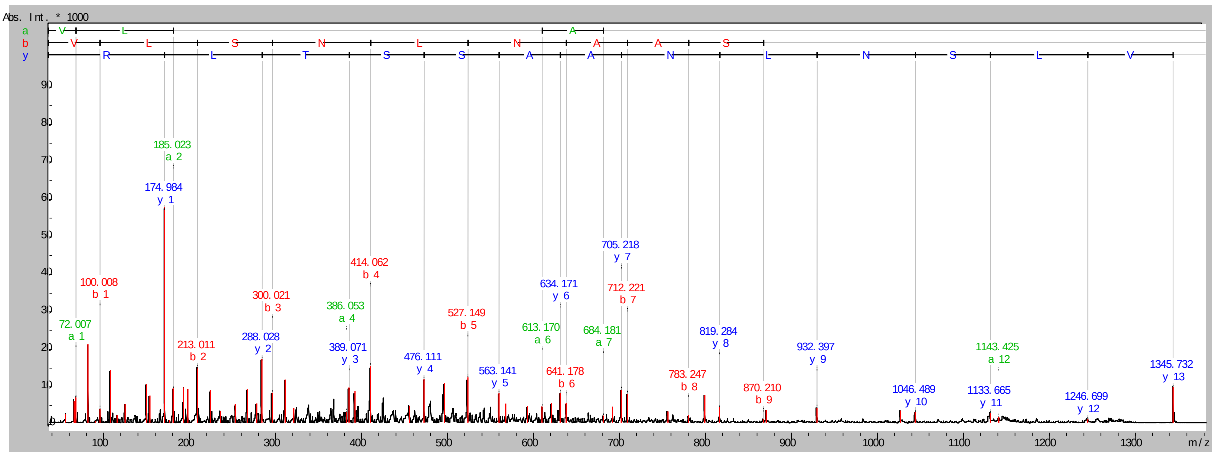

Figure S1 (cont.)

Peak 24

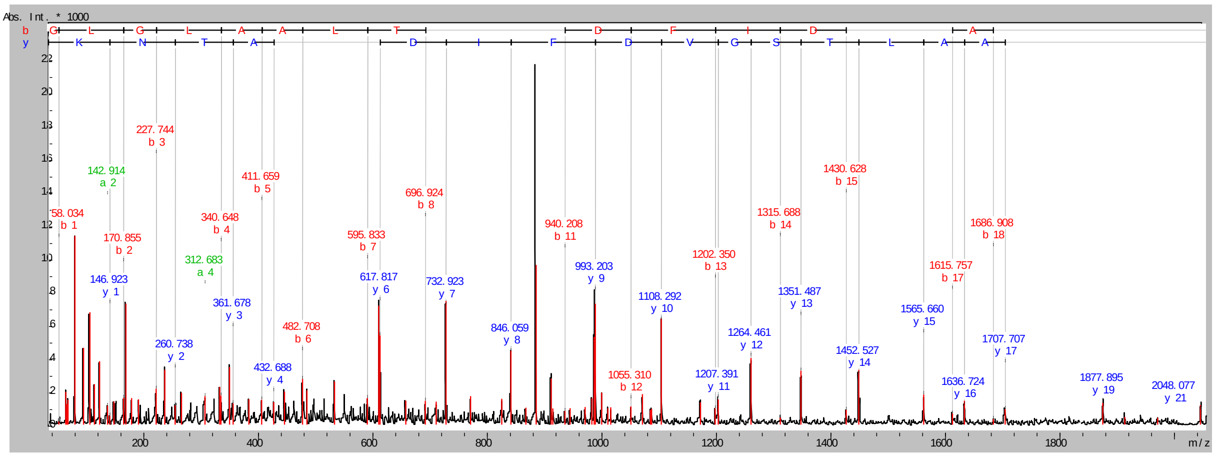

Fig. S1. Sequencing of the specific peptides of each flagellin by MALDI TOF-TOF MS^2. Sequence assignment was performed using Biotools 3.2 SR4 software with a tolerance of 0.6 Da for internal fragment assignments and 0.15 Da for parental peak. Specific peptide sequencing was used to identify each flagellin with a high confidence degree.

Table S2. Specific unique peptides from FliC1, FliC2, FliC3 and FliC4 sequenced by MALDI TOF-TOF.

Flagellins	Meas M/z	Calc MH+	Dev.(Da)	Range	Sequence
**FliC1** **637373431 NP_772483 [B. japonicum USDA 110: NC_004463]**					
peak 28	2102,15	2102,11	0.038	12 - 30	QNLLSLQSTADLLATTQER
peak 38	2336,20	2336,16	0.041	36 - 56	KVNTALDNPTNFFTAQGLDNR
**FliC2** **637373432 NP_772484 [B. japonicum USDA 110: NC_004463]**					
peak 5	1359,81	1359,76	0.046	670 - 682	VLTNLNAASSTLR
peak 14	1646,95	1646,87	0.078	683 - 698	SEASSLGSNLSVVQIR
peak 22	1817,05	1816,95	0.103	318 - 336	ALGLTTSTGAGNATVNVNR
**FliC3** **637373433 NP_772485 [B. japonicum USDA 110: NC_004463]**					
peak 15	1660,97	1660,89	0.082	683 - 698	SEASSLGSNLTIVQVR
peak 25	2060,14	2060,10	0.042	12 - 30	QNLLSLQSTADLLATTQSR
peak 35	2311,14	2311,10	0.041	36 - 56	SVNSALDNPTNFFTAQSLDNR
**FliC4** **637373434 NP_772486 [B. japonicum USDA 110: NC_004463]**					
peak 4	1345,79	1345,74	0.049	670 - 682	VLSNLNAASSTLR
peak 24	2048,12	2048,07	0.054	649 - 669	GLGLAALTSGVDFIDNAATNK

Some trajectories are difficult to classify. The complexity of the trajectories observed in this work prevented us to classify them without a quantitative criterion. Examples of trajectories that contained different features and could not be unambiguously classified are presented in Supplementary Fig. S2.

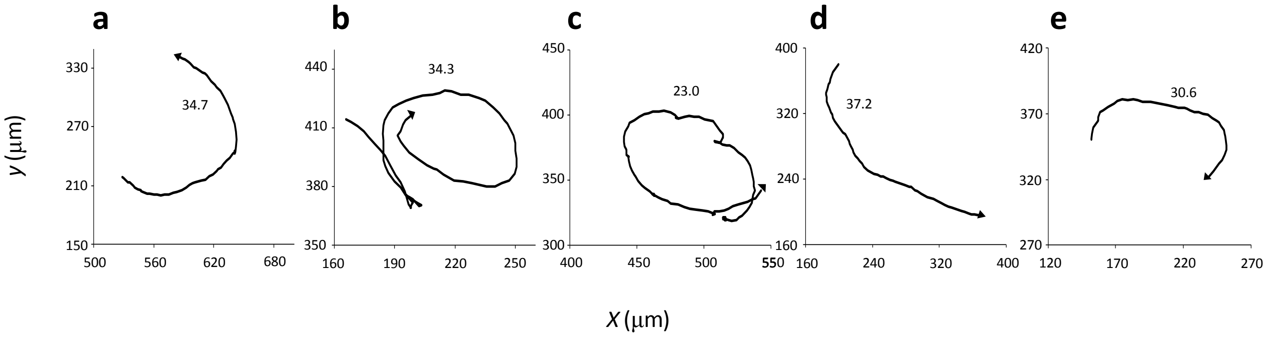

Fig. S2. Examples of trajectories difficult to classify in the classes defined in the main article: curved (**a**), linear with RR changes in direction and then circular (**b**); circular with an RRF change in direction (**c**); wide open curve (**d**); curved with a linear stretch in the middle (**e**). Coordinate axis values correspond to those of Fig. 2**a**-**d** in the main text. The numbers next to the trajectories indicate the mean speeds in micrometers per second (μm·s^{-1}).

Values of Trajectory Index (*TRAIN*) for the trajectories shown in the main article. To better illustrate how *TRAIN* was calculated, we present in Supplementary Table S3 the values of all the variables that gave rise to *TRAIN* in the trajectories shown in Figs. 2, 4 and S2. In this way, the reader may contrast the numerical values of *NGDR*, *s*, *<cosα>* and *c* against the trajectories to appreciate the extent to which *TRAIN* represents their shapes.

Table S3. *TRAIN* calculation of the trajectories exemplified in this work*

Trajectory	*TRAIN* variables				*TRAIN*
	NGDR	*s*	*<cosα>*	*c*	
Circular (2**e**)	0.13	1.18	0.95	0	1.09
Circular (4**a**)	0.12	0.77	0.98	0	1.19
Linear (2**f**)	0.97	0.34	0.95	0	1.99
RRF (2**h**)	0.20	1.91	0.89	5	0.60
RRF (4**d**)	0.13	4.56	0.80	7	0.21
RRF (4**g**)	0.60	2.78	0.90	2	1.06
RR (2**g**)	0.55	6.17	0.86	7	0.36
Rough (2**i**)	0.54	1.25	0.23	29	0.46
Diffusive (2**j**)	0.12	6.14	0.32	49	$2{\cdot}10^{-6}$
Curved (S2**a**)	0.51	0.72	0.98	0	1.62
Circular/RR (S2**b**)	0.10	3.80	0.92	3	0.78
Circular/RRF (S2**c**)	0.11	4.17	0.91	3	0.76
Wide open curve (S2**d**)	0.85	0.34	0.98	0	1.94
Curved/linear (S2**e**)	0.56	0.91	0.97	0	1.59

$*TRAIN = NGDR^{s} + \langle cos\alpha \rangle^{c}$

More images of Δ*lafA* cells from soft agar plates with viscosity at 80 mPa s. These transmission electron micrographs show the reproducibility of the flagellar sizes and disposition with respect to the cell body.

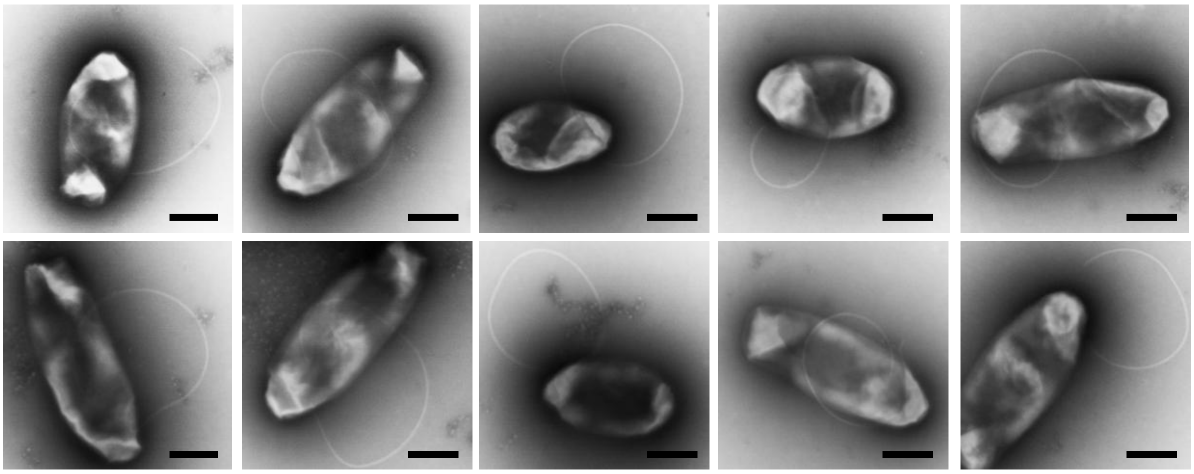

Fig. S3. Ten Δ*lafA* cells with short and curved subpolar flagella, often surrounding the cell body (scale bar: 0.5 µm).

References

60. Watt, S. A., Patschkowski, T., Kalinowski, J. & Niehaus, K. Qualitative and quantitative proteomics by two-dimensional gel electrophoresis, peptide mass fingerprint and a chemically-coded affinity tag (CCAT). *J. Biotechnol.* **106**, 287-300; DOI:10.1016/j.jbiotec.2003.07.014 (2003)
